# Selecting the quality of mule duck fatty liver based on near-infrared spectroscopy

**DOI:** 10.1186/1297-9686-46-38

**Published:** 2014-06-10

**Authors:** Christel Marie-Etancelin, Zulma G Vitezica, Laurent Bonnal, Xavier Fernandez, Denis Bastianelli

**Affiliations:** 1 UMR1388 Génétique, Physiologie et Systèmes d’Elevage, INRA, F-31326 Castanet-Tolosan, France; 2 UMR1388 Génétique, Physiologie et Systèmes d’Elevage, Université de Toulouse INPT ENSAT, F-31326 Castanet-Tolosan, France; 3 UMR1388 Génétique, Physiologie et Systèmes d’Elevage, Université de Toulouse INPT ENVT, F-31076 Toulouse, France; 4 UMR112 Systèmes d’Elevage Méditerranéens Et Tropicaux, CIRAD, Montpellier F34 398, France

## Abstract

**Background:**

“Foie gras” is produced predominantly in France and about 90% of the commercialized product is obtained from male mule ducks. The melting rate (percentage of fat released during cooking) is the main criterion used to determine the quality of “foie gras”. However, up to now the melting rate could not be predicted without causing liver damage, which means that selection programs could not use this criterion.

**Methods:**

Fatty liver phenotypes were obtained for a population of over 1400 overfed male mule ducks. The phenotypes were based on two types of near-infrared spectra (on the liver surface and on ground liver) in order to predict the melting rate and liver composition (ash, dry matter, lipid and protein contents). Genetic parameters were computed in multiple traits with a “sire-dam” model and using a Gibbs sampling approach.

**Results:**

The estimates for the genetic parameters show that the measured melting rate and the predicted melting rate obtained with two near-infrared spectrometer devices are genetically the same trait: genetic correlations are very high (ranging from +0.89 to +0.97 depending on the mule duck parental line and the spectrometer) and heritabilities are comparable. The predictions based on the spectra of ground liver samples using a laboratory spectrometer correlate with those based on the surface spectra using a portable spectrometer (from +0.83 to +0.95 for dry matter, lipid and protein content) and are particularly high for the melting rate (higher than +0.95). Although less accurate than the predictions obtained using the spectra of ground liver samples, the phenotypic prediction of the melting rate based on surface spectra is sufficiently accurate to be used by “foie gras” processors.

**Conclusions:**

Near-infrared spectrometry is an efficient tool to select liver quality in breeding programs because animals can be ranked according to their liver melting rate without damaging their livers. Thus, these original results will help breeders to select ducks based on the liver melting rate, a crucial criterion that defines the quality of the liver and for which there was previously no accurate predictor.

## Background

France is the main producer of ‘Foie Gras’ and commercializes about 73% of all fatty liver sold in the world. The most valuable product in these production systems is the fatty liver of male mule ducks, which represents 92% of the French fatty liver production. The mule duck is an intergeneric hybrid between a male Muscovy duck (*Cairina moschata*) and a female common duck (*Anas platyrhyncos*). Technically, the quality of fatty liver is measured by the percentage of fat loss during cooking, which is called “melting rate” and is expected to be as low as possible. The processing yield represents the amount of product remaining after cooking, i.e. 100% minus the melting rate. There is a strong variability in the processing yield of fatty liver from mule ducks even when overfeeding and processing conditions are controlled. This variability is a problem for the industry, mainly because a maximum fat loss during cooking of 30% of the raw liver weight is laid down by French law (Journal Officiel de la République Française, 1993).

The melting rate (MR) and biochemical composition of fatty liver are not easily measured in commercial conditions. Both require either the partial or total destruction of the liver. Traditionally, fatty liver weight has been used as a predictor of MR for the industrial sorting of fatty livers due to its negative relationship with MR. However selection programs cannot be based on this parameter because it would lead to reduced liver weights. In addition, liver weight explains only 14% of the phenotypic variation of MR so its use for selection would be quite inefficient [1]. Recently, in addition to the use of the weight of fatty livers, biochemical traits, such as dry matter and protein content, were measured and used to predict the processing yield [1]. Such biochemical measures were found to be more accurate predictors of the processing yield than the liver weight alone. However, the predictive ability of the model remained low (R^2^ = 0.43) even when both the biochemical composition and the weight of fatty liver were taken into account, and the biochemical measures required partial destruction of the liver.

To date, selection on the melting rate has never been implemented, although 90% of the price of fatty liver is based on this trait. Neither fatty liver weight nor biochemical composition is an adequate phenotype to rank selection candidates. Under experimental conditions, the heritability estimates obtained for MR (measured in mule ducks) had intermediate values (0.19 ± 0.04) on the maternal common line and low values (0.09 ± 0.03) on the paternal Muscovy line [2]. Thus, selection for MR would be interesting for both parental mule duck lines.

Near-infrared spectroscopy (NIRS) is an analytical technique based on the absorption of infrared light by organic matter. Since absorption is linked to the chemical composition of samples, it can be predicted after a calibration phase. NIRS is widely used to predict the composition of animal products [3] and for phenotypic prediction [4-13] in pigs, cows, chicken and waterfowl. Researchers have investigated dynamic (at-line or on-line) NIRS applications to qualify a sample *in situ* during technological processing (either for meat quality for pork [14], beef [15] or milk coagulation ability [16]) or offline NIRS applications for genetic variability estimates (either for meat composition or quality in pigs [17,18], rabbits [19] and cows [20,21] or for milk coagulation properties [22-24]). For all these experiments, NIRS appeared as a suitable alternative to classical analytical procedures. Generally, the NIRS equipment used in these studies were laboratory spectrometers measuring ground samples. Portable spectrometers with a separate probe for spectrum acquisition directly on the product surface are an alternative but their use is still limited [8]. The aim of this study was to compare genetic parameter estimates (in both parental lines) for the melting rate of duck fatty liver as measured by either the reference method or predicted using various NIRS technologies (offline on ground samples in the laboratory or at-line on intact livers) in order to select parental lines.

## Methods

The present study was carried out in agreement with the French National Guidelines for the care and use of animals for research purposes (Certificate no. 7740, Ministry of Agriculture and Fish Products, Paris, France).

### Animals

A total of 1552 male mule ducks were hatched over a two-year period. For both years, about 800 mule ducks were produced in two pedigree batches of 400 ducklings at the INRA Experimental Farm for Waterfowl Breeding (INRA-UEPFG, France). The mule ducks were hybrids between two experimental populations: 382 female back-cross (BC) common ducks (*Anas Platyrhynchos*) and 56 Muscovy drakes (*Cairina moschata*).

For each year and hatch, mule ducklings were bred in eight batches of about 50 animals. From hatching to 10 weeks of age, mule ducks were fed *ad libitum* with a commercial diet. From 10 to 12 weeks of age, duck were feed-restricted by 30 g/d per duck and then put back on a *ad libitum* diet at the beginning of the 12^th^ week. At 12 weeks of age, ducks were bred for 12 days in collective cages of four or five individuals and were overfed twice a day with a mix (35% corn-flour, 25% corn-grain and 40% water), in two successive series of 200 animals with two different force-feeders. At the end of the overfeeding period (92 or 94 days of age according to series), animals were slaughtered by severing the neck blood vessels after electrical stunning. Mule ducks were bled, plucked, and cooled to 4°C. Twenty four hours after slaughter, the fatty livers were extracted from the carcasses. Due to mortality (3.5%) and bleeding liver problems (0.4%), fatty livers were collected for 1492 mule ducks. Subsequent analysis was performed on livers weighing between 300 g and 830 g (1422 fatty livers) in order to be consistent with the range of weights of commercialized livers.

### Analytical measurements

On the day of liver extraction, the fatty liver melting rate was measured on all livers by a cooking test as the percentage of fat released after sterilization (65 min at 105°C) of 60 g of liver. A round slice (40 mm diameter) was removed from each raw liver and two other samples (about 20 g per sample) were ground. All samples were stored at −20°C prior to spectrometric and biochemical analyses. In addition, a subset of 198 liver samples, selected for the development of NIRS calibration equations, were analyzed using the reference biochemical laboratory methods: lipid content using cold chloroform/methanol extraction [25], dry matter after desiccation of the liver for 24 h in a drying oven at 103°C [26], ash content in a muffle furnace at 550°C [27] and protein content by wet mineralization (Kjeldhal method) [28].

### Spectral measurements

Two types of reflectance spectrometry measurements were performed. A first measurement was performed directly on the fatty liver surface immediately after liver extraction (at 24 h *post mortem*) using a portable ASD Labspec Pro spectrometer (wavelength range: 350–2500 nm) with a “contact probe” module. Six spectra were collected for each fatty liver and the average value was used for the calibration. A second set of measurements was performed in the laboratory on ground liver samples presented in quartz cells using a FOSS NIRSystems 6500 (wavelength range: 400–2500 nm). Each sample was measured three times (three different aliquots of the same sample) and the average spectrum was used for the subsequent calibration. Spectral data were processed using WINISI software (Infrasoft Int., Port Matilda, Pennsylvania, USA). The visible wavelengths of the spectrum were discarded to avoid hypersensitive models in which differences in color unrelated to liver composition and melting rate were taken into account. The wavelengths used were 800 to 2500 nm. Several statistical pre-processing steps [29] were tested such as combining the order of derivation (0, 1 or 2), varying the number of data points for smoothing and derivation (0, 5, 10, 15 or 20 data points) and mathematical treatment (normalization, detrending and multiplicative scatter correction). The best results (lowest cross-validation errors during calibration) were obtained with the first derivative (calculated on 10 data points) combined with smoothing (5 data points) on normalized spectra (SNV = standard normal variate).

The NIRS calibration database consisted of 198 liver samples that were selected in order to represent the spectral variability of the total set of ground samples measured using the FOSS spectrometer and characterized by analytical measurements previously described. For each spectrometer, the calibration equations were developed for melting rate (MR) and for dry matter (DM), ash content (AC), lipid content (Lip) and protein content (Prot) as reported by Bastianelli et al*.*[30]. In the WINISI software, the procedure of representative sample selection is based on PCA (Principal Component Analysis). The calibration equations were obtained by Partial Least Square (PLS) regression. A cross-validation was performed on five groups during the calibration process: calibration was performed on four groups and then applied to the remaining group; this process was repeated five times and the average error was calculated (SECV, Standard Error of Cross-Validation). Then the calibration equations were validated against the 1224 samples (i.e. 1422 minus 198) that were not used for calibration and formed an external dataset. Hence the predictive ability of the equation can be estimated on new samples. The resulting prediction error (SEP, Standard Error of Prediction) was calculated.

### Statistical analysis

The traits analyzed were the measured melting rate (mMR) of the liver, and liver attributes, respectively, predicted using FOSS and ASD spectrometers, such as the melting rate (pMR-FOSS and pMR-ASD, respectively), dry matter (pDM-FOSS and pDM-ASD, respectively), the ash content (pAC-FOSS and pAC-ASD, respectively), lipid content (pLip-FOSS and pLip-ASD, respectively) and protein content (pProt-FOSS and pProt-ASD, respectively). According to the Kolmogorov-Smirnov test at 5%, these 11 traits were normally distributed.

Genetic parameters were estimated by combining pedigree information from both parental populations (common and Muscovy) and from mule duck performances, using the model of Lo et al*.*[31]:

$$\left[y_{C}\right]=\left[X_{C}\right]\times \left[b_{C}\right]+\left[Z_{\mathrm{AC}}Z_{\mathrm{BC}}\right]\times \left[\begin{array}{c} u_{\mathrm{AC}}\\ u_{\mathrm{BC}} \end{array}\right]+\left[e_{C}\right]$$

where **y**_**C**_ is the vector of observations for crossbreds (1422 mule ducks); **b**_**C**_ the vector of systematic effects for the combination of year, batch and force-feeder effects (12 levels); **u**_**AC**_ (**u**_**BC**_) the additive genetic effects of the common dams (Muscovy sires) of mule ducks, as expressed in the phenotypes of the mule ducks (population C); **e**_**C**_ the vector of residual effects; and **X** and **Z** the incidence matrices relating the observations to the corresponding effects. We stress that **e**_**C**_ contains the Mendelian sampling of crossbred animals, as well as the “true” residual environmental effects. In short, the model substitutes the genetic effect of the crossbred with half the breeding value of their parents, plus a Mendelian sampling. Pedigrees were traced back up to five generations of ancestors for both parental lines and consisted of 596 animals in the common line and 201 animals in the Muscovy line. Multiple trait genetic parameters were estimated by Gibbs sampling using the program “gibbs1f90” [32]. Flat priors were used for variances and covariance. The variance and covariance components of given averages were respectively equal to 1 and 0.01. A total chain length of 100 000 iterations was run and 20 000 samples were discarded as burn-in.

The heritability estimates are the ratio between the paternal variance (versus the maternal variance) and the total variance for the Muscovy parental line (versus the common parental line). These estimates cannot be considered as “real” heritabilities of mule ducks but rather as “partial” heritabilities.

## Results

### Measured and predicted melting rates

The characteristics of calibration equations for the melting rate are reported in Table 1. For the FOSS spectrometer, the coefficient of determination (R^2^) of the calibration equation was high for the melting rate (0.87). The prediction error, estimated by SECV, was also quite high (6.07%) but sufficient to rank samples given the high variability of the melting rate in the population (SD = 14.7%). With the ASD spectrometer, the R^2^ value was only 0.02 points lower than with the FOSS spectrometer; the SECV value was therefore slightly higher (7%). The external validation process on 1224 samples confirmed the predictive ability of NIRS with SEP values that were close to SECV values for both spectrometers. An interesting point is that the measurements performed with the ASD spectrometer on intact livers taken immediately after liver extraction resulted in a good prediction of the melting rate. This result is consistent with the results obtained for the biochemical composition of livers in the same conditions [30].

**Table 1:** Characteristics of calibration equations for measured melting rate (mMR) obtained with FOSS and ASD spectrometers

	**Calibration N = 198**					**Validation N = 1224**		
	**Mean**	**SD**	**SEC**	**R**^**2**^	**SECV**	**Mean**	**SD**	**SEP**
FOSS	35.6	14.7	5.34	0.87	6.07	39.2	11.9	6.56
ASD	35.6	14.7	5.69	0.85	6.52	39.2	11.9	7.12

The FOSS and ASD spectrometers provided similar estimated heritabilities within the parental lines (Table 2) since the differences between spectrometers (from 0.01 to 0.02) were lower than credibility intervals (from 0.03 to 0.04). The melting rate heritabilities estimated using NIRS ranged from 0.18 to 0.20 in the common line and from 0.11 to 0.12 in the Muscovy line. These results are fully comparable with the heritability estimates based on measured melting rates which were equal to 0.20 ± 0.03 in the common line and 0.10 ± 0.03 in the Muscovy line. It is worth noting that heritability values tended to be lower in the Muscovy line than in the common line.

**Table 2:** Heritabilities (and standard deviation) for measured and predicted melting rates using two NIRS spectrometers (ASD, FOSS) in both parental lines

	**Common line**	**Muscovy line**
mMR	0.20 (0.03)	0.10 (0.03)
pMR- FOSS	0.20 (0.03)	0.12 (0.04)
pMR-ASD	0.18 (0.03)	0.11 (0.04)

mMR = measured melting rate; pMR-FOSS = melting rate predicted by FOSS; pMR-ASD = melting rate predicted by ASD.

The genetic correlations between the different melting rates (measured and predicted with FOSS and ASD spectrometers) are in Table 3. For the common line, correlations ranged from +0.89 to +0.95 and for the Muscovy line, all estimates were equal to +0.97. Therefore, it is reasonable to assume that the melting rate traits measured or predicted (either with ground samples and a FOSS spectrometer or on intact samples at slaughter with the ASD spectrometer) are genetically the same trait. In addition, the ranking of the selection candidates was the same (Spearsman’s rank correlations greater than 0.90), regardless of the melting rate trait used to estimate breeding values.

**Table 3:** Genetic correlations (and standard deviation) for different melting rates in both parental lines

**Line**		**pMR-FOSS**	**pMR-ASD**
Common	mMR	+0.89 (0.03)	+0.93 (0.03)
	pMR-FOSS		+0.95 (0.03)
Muscovy	mMR	+0.97 (0.02)	+0.97 (0.04)
	pMR-FOSS		+0.97 (0.03)

mMR = measured melting rate; pMR-FOSS = melting rate predicted by FOSS; pMR-ASD = melting rate predicted by ASD.

### Biochemical parameters

The heritabilities estimated for biochemical parameters (Table 4) using FOSS and ASD spectrometers were lower than those for the melting rate in both parental lines, but differences were particularly pronounced in the common line. The heritability estimates for the liver ash content were null (values of 0.01 ± 0.01 regardless of the line and spectrometer). As observed for the melting rate, heritability values tended to be lower in the Muscovy line than in the common line, whatever the trait.

**Table 4:** Heritabilities and genetic correlations (and their standard deviation) for liver composition traits predicted using two NIRS spectrometers (ASD, FOSS) and melting rates in both parental lines

**Line**		**pDM-FOSS**	**pDM-ASD**	**pAC-FOSS**	**pAC-ASD**	**pLip-FOSS**	**pLip-ASD**	**pProt-FOSS**	**pProt-ASD**	**mMR**
**Common**	pDM-FOSS	0.12 (0.03)	+0.95 (0.03)							+0.90 (0.06)
	pDM-ASD		0.15 (0.03)							+0.89 (0.05)
	pAC-FOSS			0.01 (0.01)	+0.94 (0.03)					−0.95 (0.05)
	pAC-ASD				0.01 (0.01)					−0.87 (0.05)
	pLip-FOSS					0.14 (0.03)	+0.87 (0.05)			+0.84 (0.05)
	pLip-ASD						0.12 (0.03)			+0.81 (0.06)
	pProt-FOSS							0.13 (0.03)	+0.93 (0.03)	−0.89 (0.04)
	pProt-ASD								0.11 (0.03)	−0.91 (0.07)
**Muscovy**	pDM-FOSS	0.08 (0.03)	+0.96 (0.03)							+0.90 (0.08)
	pDM-ASD		0.09 (0.03)							+0.83 (0.11)
	pAC-FOSS			0.01 (0.01)	+0.81 (0.10)					−0.88 (0.09)
	pAC-ASD				0.01 (0.01)					−0.82 (0.11)
	pLip-FOSS					0.07 (0.03)	+0.83 (0.10)			+0.95 (0.06)
	pLip-ASD						0.07 (0.03)			+0.90 (0.09)
	pProt-FOSS							0.07 (0.03)	+0.87 (0.07)	−0.96 (0.04)
	pProt-ASD								0.07 (0.03)	−0.88 (0.09)

pDM-FOSS: dry matter predicted by FOSS; pDM-ASD: dry matter predicted by ASD; pAC-FOSS: ash content predicted by FOSS; pAC-ASD: ash content predicted by ASD; pLip-FOSS: lipid content predicted by FOSS; pLip-ASD: lipid content predicted by ASD; pProt-FOSS: protein content predicted by FOSS; pProt-ASD: protein content predicted by AS; mMR: measured melting rate.

The genetic correlations between a biochemical trait predicted with the FOSS spectrometer and the same trait predicted with the ASD spectrometer are in Table 4. For the common line, correlations ranged from +0.87 to +0.95 and for the Muscovy line estimates were a little smaller (from +0.81 to +0.96). Except for dry matter, the correlations between both predictions for biochemical traits were lower than correlations for liver melting rate (0.95 and 0.97 for the common and Muscovy lines, respectively).

The genetic correlations between the measured melting rate and the biochemical traits predicted using either the FOSS or the ASD spectrometer (Table 4) were high and similar for both parental lines, ranging in absolute value from 0.81 to 0.95 for the common line and from 0.82 to 0.96 for the Muscovy line.

## Discussion

The R^2^ value obtained here for the melting rate predicted with the FOSS spectrometer is high (0.87), ranging between the R^2^ value for lipid (0.93) and protein contents (0.78) [30]. It is higher than the R^2^ value for milk processing traits predicted using Mid-InfraRed (MIR) spectroscopy [22], which range from 0.61 to 0.69 for the rennet coagulation time and from 0.46 to 0.52 for curd firmness. Different factors contribute to the lower performance of the ASD spectrometer for fatty liver predictions (R^2^ = 0.85) compared to the FOSS spectrometer: the conditions of spectrum collection (in particular a more constant ambient temperature in the laboratory than in the slaughterhouse), artefacts due to the presence of condensed water on the liver surface, and finally the optic fiber probe used with the ASD spectrometer that might decrease its accuracy [18]. Nevertheless, the differences in the accuracy of the two spectrometer techniques is lower than that reported by Marie-Etancelin et al*.*[33] for the composition of duck breast meat. Some experiments conducted on the use of NIRS on the surface of beef meat to predict quality traits [8] led to the conclusion that it was possible to predict meat color but that NIRS correlations for physical or sensory traits were too low and resulted in irrelevant predictions. In our study, and in contrast with Prieto et al. [8] or to a lesser extent with Cecchinato et al*.*[22], the processing yield obtained with the ASD spectrometer on the surface of fatty liver is sufficient to predict phenotypic values for the melting rate. The melting rate seems to be an exception among processing traits because it is predicted correctly using a NIRS surface measurement. This could be due to the correlation of MR with the biochemical composition of fatty liver (R^2^ of 0.58 between MR and fat content).

Genetic parameters of mule fatty liver traits were estimated for each parental population and we observed that, whatever the trait, the standard deviations of genetic correlations were higher in the Muscovy line than in the common line. A similar trend was observed for all traits studied in the same dataset [2]. The smaller number of animals in the Muscovy pedigree (compared to the common line) could explain the lower accuracy of the correlations. In addition, heritability values were clearly higher in the common population compared to the Muscovy line (with differences ranging from 0.02 to 0.10 points depending on the trait) but the genetic correlations were similar. These differences in heritability of traits depending on the parental line have already been reported by [34] for carcass and muscle weight and by [2] for the vast majority (but not all) of the 37 mule ducks traits studied.

Considering that the mule ducks were full-sibs on the dam’s side and a mix between full- and half-sibs on the sire’s side, the partial heritability estimates should be multiplied by 2 in the common population and by a value between 2 and 4 in the Muscovy population. Thus, the differences in heritability between the parental populations were greatly reduced. Nevertheless, as discussed in [2], two hypotheses can be proposed: either there is a real difference between the genetic determinism of the two parental lines, or the maternal or family effect is absorbed in the additive genetic effect of the female duck resulting in artificially overestimated heritabilities in the maternal common line.

To compare our heritability estimates with previously published values, we must consider that, due to the “sire-dam” model used, the total genetic variability of mule traits was divided into the paternal and the maternal lines, the sire and the dam accounting for a ratio between a quarter to a half of the additive variance of the trait. Since additive genetic correlations between predicted and measured melting rates were higher than 0.89, we concluded that NIRS is a useful criteria to select MR in breeding programs. Studies on the genetic determinism of quality traits predicted by NIRS are scarce, and are mostly related to the composition of meat and very rarely to the processing capacity of products. Only Cecchinato et al*.*[21] published results for predicting cooking losses of beef meat with NIRS, and concluded that NIRS could not be used to predict traits because R^2^ was low and the heritability and genetic correlations with physical analysis were close to zero. However, Cecchinato et al*.*[22] demonstrated that the rennet coagulation time of cow milk could be selected using MIR spectra. Our genetic study on the melting rate of fatty liver - the main processing trait for the fatty liver industry - is therefore original and moreover leads to relevant results particularly for the selection of ducks.

The genetic correlation between the predictions of the melting rate with the two spectrometers is very high (>0.95); the correlations with the measured melting rate are also very strong. Thus, we conclude that predictions using the ASD spectrometer, which preserve the fatty liver integrity and is the only operational spectrometry technique that can be used under industrial conditions, will enable effective selection based on the fatty liver melting rate. Moreover, the genetic correlations between the measured melting rate and each of the predicted biochemical parameters are equal or lower than the genetic correlations between the measured melting rate and its NIRS predictions. If all ‘biochemical’ phenotypes associated (correlated) with measured melting rate were combined in a single predictor of melting rate, the accuracy of this predictor could be obtained using the Selection Index theory [35]. This accuracy reached +0.92 for the common line and +0.91 for the Muscovy line using the biochemical measurements [33]. In the present study, accuracies using ASD spectrometer measures were a little higher: +0.93 for the common line and +0.97 for the Muscovy line. Moreover, developing NIRS prediction equations to predict the biochemical analyses and estimating the accuracy of MR prediction are more complex and expensive than developing NIRS prediction equations directly from melting rate measurements.

## Conclusions

This study shows that the melting rate predicted on undamaged products using an at-line ASD spectrometer is similar to that obtained on ground samples using an offline FOSS spectrometer. The NIRS predictions and the measured melting rate are the same trait. From a genetic point of view, selection on the fatty liver melting rate is now possible using NIRS technology. In breeding programs, the ASD spectrometer seems to be a more appropriate support because spectra are collected directly in the slaughterhouse and the fatty liver, a valuable product, is not damaged. From a commercial point of view, NIRS-based predictions can be used to evaluate melting rates and adapt the process to the quality of fatty livers while maintaining liver integrity.

## Competing interests

The authors declare that they have no competing interests.

## Authors’ contributions

All authors were involved in the conception of the study. CME designed the experiment with the participation of XF. CME obtained the funds required to perform the study. CME and ZGV performed the genetic analyses and drafted the manuscript. DB and LB collected the spectra and computed the NIRS predictions. All authors read and approved the final manuscript.
